# Comparative genome mapping of the deer mouse (*Peromyscus maniculatus*) reveals greater similarity to rat (*Rattus norvegicus*) than to the lab mouse (*Mus musculus*)

**DOI:** 10.1186/1471-2148-8-65

**Published:** 2008-02-26

**Authors:** Clifton M Ramsdell, Adrienne A Lewandowski, Julie L Weston Glenn, Paul B Vrana, Rachel J O'Neill, Michael J Dewey

**Affiliations:** 1 Department of Genetics and the Carolina Center for Genome Sciences, University of North Carolina, Chapel Hill, North Carolina 27599, USA; 2 Peromyscus Genetic Stock Center, Department of Biological Sciences, University of South Carolina, Columbia, SC 29208, USA; 3 Department of Biological Chemistry, School of Medicine, University of California Irvine, Irvine, CA 92799-1700, USA; 4 Department of Molecular and Cell Biology, University of Connecticut, Storrs 06269, USA

## Abstract

**Background:**

Deer mice (*Peromyscus maniculatus*) and congeneric species are the most common North American mammals. They represent an emerging system for the genetic analyses of the physiological and behavioral bases of habitat adaptation. Phylogenetic evidence suggests a much more ancient divergence of *Peromyscus *from laboratory mice (*Mus*) and rats (*Rattus*) than that separating latter two. Nevertheless, early karyotypic analyses of the three groups suggest *Peromyscus *to be exhibit greater similarities with *Rattus *than with *Mus*.

**Results:**

Comparative linkage mapping of an estimated 35% of the deer mouse genome was done with respect to the Rattus and Mus genomes. We particularly focused on regions that span synteny breakpoint regions between the rat and mouse genomes. The linkage analysis revealed the Peromyscus genome to have a higher degree of synteny and gene order conservation with the Rattus genome.

**Conclusion:**

These data suggest that: 1. the *Rattus *and *Peromyscus *genomes more closely represent ancestral Muroid and rodent genomes than that of *Mus*. 2. the high level of genome rearrangement observed in Muroid rodents is especially pronounced in *Mus*. 3. evolution of genome organization can operate independently of more commonly assayed measures of genetic change (e.g. SNP frequency).

## Background

The cricetid genus *Peromyscus *constitutes the most abundant and speciose group of North American mammals. Though superficially similar in appearance to rats and mice, deer mice represent a more distantly related lineage. Mouse and rat are thought to have diverged from each other ~10–12 million years ago (mya) while they last shared a common ancestor with the deer mouse (*P. maniculatus*) lineage ~25 mya [1]. The *P. maniculatus *species complex is a series of semi-interfertile populations spanning nearly every habitat on the continent and is consequently an emerging tool for the study of natural mammalian genetic variation. Facilitating such research is the existence of captive stocks derived from individual populations. Utilizing two of these stocks, we have developed a comparative genomic map for the deer mouse to further research of this genus and to provide insight into the genome rearrangements seen in rats, mice, and other mammals.

Comparative genomic analyses can reveal substantial amounts of information about the biology and evolution of species and are one of the keys to deciphering the roles that genomic structure and organization play in areas such as development, gene expression, and speciation. These analyses, however, are limited to portions of the genomes that have been mapped in all the species being compared and may be compromised by uncertainty of gene orthology and order between any two species.

Although whole-genome sequences are available for many species, proper genome annotation is difficult and typically requires additional resources (e.g. meiotic linkage and radiation hybrid cell maps) [2]. As a result, both cytogenetic methods and genetic linkage mapping are still essential tools for the analysis of genomic organization. While cytogenetic methods are effective for discerning large regions of chromosomal homology and conserved synteny, linkage maps are able to detect rearrangements of gene order within these fragments and pinpoint the locations of synteny breakpoints. Such detailed genomic comparisons require ordered linkage maps that include orthologous Type I (gene coding) loci to provide landmarks that can be identified in the genomes of multiple species. Comparative analyses using such a combined approach may reveal many more chromosomal rearrangements and novel synteny groups.

Two of the most complete mammalian genomic maps are associated with the most used biomedical models, the rat (*Rattus norvegicus*) and mouse (*Mus musculus*), which both belong to the rodent family Muridae. Rodentia is the largest mammalian order, containing > 2000 of the ~4600 recognized species and the murids constitute the majority of these [3]. Murid genomes analyzed to date not only show more rapid nucleotide mutation rates [1,4] but also higher rates of chromosomal rearrangement than other mammals. Murid chromosomal divergence rates are estimated to be one rearrangement per million years; ten times the rate for most mammalian genomes [5,6]. Furthermore, such events are punctuated over time rather than having a steady-state mutation pattern [7]. This elevated rate of rearrangement has resulted in greater karyotypic divergence between rat and mouse than between much more distantly related species (e.g., humans vs. domestic cats) [8] and has hampered reconstructions of ancestral rodent and mammalian genomes [9-12]. As a result, interpreting the evolutionary trajectory of chromosome segments between these model organisms and humans has proven difficult [13]. An outgroup to the two mapped murid genomes that is less divergent than human could alleviate these problems by aiding in the construction of a more accurate ancestral rodent genome.

Here we describe the initial results of the first intermediate-density comparative genomic map for the deer mouse covering an estimated 35% of the deer mouse genome. These data suggest the deer mouse genomic organization more closely resembles that of rat than mouse, despite the much more recent common ancestor shared by the latter two species. Considered with cytogenetic data [14] and ancestral karyotype reconstructions [10,12], our analysis further suggests that the deer mouse and *Rattus *genomes have undergone fewer large scale rearrangements than *Mus*.

## Results and Discussion

### Design

We employed a standard backcross design for these studies utilizing *P. maniculatus bairdii *stock derived from Washtenaw Co. MI (BW) and *P. polionotus *stock derived from Ocala Nat'l forest in Florida (PO). While neither population is completely inbred, both originated from a limited number of founders and have been maintained as closed colonies. Thus, identifying fixed differences between the two (e.g., SNPs) was typically not difficult.

Map construction for *P. maniculatus *was conducted using both the *Rattus *and *Mus *maps as references [15,16] and using assays designed to span rat-mouse synteny breakpoints. We use the term breakpoint when referring to a break between linkage groups as defined by Pevzner and Tesler [13]. In all, we have genotyped 103 Type I gene markers from 18 different *Rattus *chromosomes on our backcross panels. Table 1 presents the complete list of markers used in our mapping study, their respective locations in the mouse and rat genomes, and the source of the primer sequences. The figures presented here though, focus on the markers around the breakpoint regions between *Mus *and *Rattus *genomes that are informative for this comparative analysis.

**Table 1:** Markers utilized in this study, their positions in the *Rattus *and *Mus *genomes, and their source.

Marker	*Rattus *Chr.	*Rattus *Mb	*Mus *Chr.	*Mus *Mb	Marker Source
Plagl1	1	7.9	10	12.8	This study
Clptml1	1	30.5	13	74.1	PM_BWp0019B05
Mas1	1	42.1	17	11.7	[36]
Pacrg	1	44.4	17	10.3	PM_BWt0020A08
Tcp10b	1	47.6	17	6.8	[32]
Usp29	1	65.4	7	5.9	This study
Zfp574	1	80.5	7	20.3	PM_BWt0030D07
Hnrpl	1	84.0	7	24.2	PM_BWt0015D0f
Q8C5Y2	1	91.8	7	43.2	PM_BWt0034E03
Myod1	1	96.9	7	46.3	This study
Ube3a	1	110.8	7	59.1	PM_BWt0035G08
Rab6ip1	1	167.6	7	109.7	PM_BWt0031E09
H19	1	202.8	7	137.0	[35]
Prdx5	1	209.7	19	7.0	PM_BWt0010H02
Prpf19	1	213.6	19	11.0	PM_BWt0050D11
Xpnpep1	1	259.4	19	53.1	PM_BWt0029G03
Spz1	2	22.8	13	93.7	PM_BWt0006E03
Car1	2	88.1	3	14.7	PM_BWt0033B03
Tloc1	2	116.3	3	30.1	Contig [t0041H10]
Golph4	2	166.7	3	75.1	PM_BWt0011G08
Atp1a1	2	196.6	3	101.0	PM_BWp0001H12
Unc5c	2	239.4	3	140.4	PM_BWt0025F04
Fbxw5	3	3.7	2	25.4	Contig PM_BWt0016A08U
Clp1	3	67.9	2	84.5	PM_BWt0033G11
Chst1	3	77.7	2	92.4	PM_BWp0021H02
Apip	3	88.3	2	102.9	PM_BWt0003D08
Thbs1	3	103.9	2	117.8	PM_BWp0019F04
Adra1d	3	119.2	2	131.1	[32]
Dpm1	3	159.4	2	167.9	PM_BWt0028C07
Srpk2	4	6.9	5	23.0	Contig PM_BWt0030E08U
Sri	4	*21.6 (Celera)*	5	8.1	PM_BWt0020G07
Ccdc132	4	28.3	6	3.5	PM_BWt0038C06
1700016G05Rik	4	68.4	6	40.4	Contig [t0039B08]
Gabarapl1	4	167.0	6	129.5	PM_BWt0019H08
Tram1	5	4.8	1	13.7	PM_BWt0037H08
Oprk1	5	14.0	1	5.6	This study
Ube2j1	5	49.3	4	33.3	PM_BWp0021E08
Rnf20	5	66.4	4	49.5	PM_BWt0013E09
Ubxd5	5	152.9	4	133.4	PM_BWt0007G04
Spata21	5	160.0	4	140.0	PM_BWt0027G03
Mmel1	5	171.7	4	153.7	PM_BWt0028B02
Sos1	6	3.3	17	78.2	[40]
Ppp1cb	6	24.1	5	32.7	PM_BWp0006A12
Preb	6	25.4	5	31.2	PM_BWt0037F10
Dnmt3a	6	26.8	12	3.8	This study
Allc	6	46.4	12	29.1	PM_BWt0049G04
4930504H06Rik	6	51.7	12	34.0	PM_BWt0033A03
Clec14a	6	78.9	12	59.2	PM_BWp0007A11
Pygl	6	92.3	12	71.1	PM_BWp0007B06
Pcnx	6	105.7	12	82.8	Contig [t0010D02]
1700001K19Rik	6	135.3	12	111.1	Contig [t0029G05]
Il23a	7	1.6	10	128.0	[34]
Stk11	7	11.1	10	79.5	PM_BWp0005E08
Hsp90b1	7	23.4	10	86.1	PM_BWp0013F08
Phkd1l1	7	80.3	15	44.3	Contig [T0025f09]
C920006C10Rik	7	103.2	15	65.6	PM_BWt0015A02
Adck5	7	114.7	15	76.4	PM_BWt0028A12
Mut	9	15.8	17	40.4	Contig [t0024G12]
Col9a1	9	22.9	1	24.4	[31]
Col3a1	9	44.3	1	45.6	[31]
Fn1	9	70.9	1	71.9	[32]
Lama1	9	107.1	17	65.5	[32]
Btbd12	10	11.8	16	3.7	PM_BWt0019F01
Gbl	10	13.7	17	22.3	PM_BWp0005A03
Hba	10	15.6	11	32.2	[31]
Canx	10	35.9	11	49.9	PM_BWp0001C07f
Sparc	10	40.9	11	55.0	[32]
Trp53	10	56.4	11	69.2	M. McLachlan (pers. comm.)
Mpo	10	76.1	11	87.4	[32]
HoxB	10	85.1	11	96.0	[32]
Gast	10	89.3	11	100.0	[33]
Myl4	10	93.7	11	104.4	[32]
Scn4a	10	95.8	11	106.1	[31]
Sstr2	10	96.2	11	113.3	[33]
P4hb	10	110.0	11	120.2	[32]
Arvcf	11	846.0	16	18.3	PM_BWp0007D05
Flt1	12	7.9	5	146.5	PM_BWp0007C04
Lrch4	12	19.7	5	136.6	Contig PM_BWt0042B01
Piwil1	12	28.9	5	127.9	Contig PM_BWt0011G03
Mapkapk5	12	36.1	5	121.8	PM_BWt0019E09
Bcl2	13	12.7	1	106.5	[32]
Glul	13	65.9	1	155.7	[33]
Acbd3	13	96.7	1	180.7	PM_BWP0009B01
Afp	14	19.1	5	89.8	PM_BWp0002H11
Pcdcl2	14	34.2	5	75.6	PM_BWt0012A04
Hip2	14	45.4	5	64.3	PM_BWt0028D02
Xbp1	14	86.2	11	5.4	PM_BWp0012A03
Igfbp1	14	88.0	11	7.1	[32]
Grb10	14	92.8	11	11.8	[17]
Ugp2	14	102.1	11	21.2	PM_BWp0009C07
Ecd	15	4.2	14	19.1	PM_BWt0026E07
Cma1	15	34.1	14	50.5	[31]
Itm2b	15	54.0	14	72.1	PM_BWp0020C01
9130404D08Rik	16	19.9	8	72.9	PM_BWt0040G05
Spata4	16	35.3	8	56.1	contig [t0034B10]
Mtus1	16	54.6	8	39.9	contig [too42G11]
Adam3	16	71.7	8	23.4	contig [t0035F12]
Sec61a2	17	83.5	2	5.8	PM_BWt0014H11U
5430411K18Rik	18	74.9	18	78.1	PM_BWt0031G08
Hps4	19	14.4	5	112.6	PM_BWp0006B12
Phkb	19	22.3	8	85.1	PM_BWt0031G11
Elmod2	19	26.2	8	82.6	PM_BWt0036H11

* Mb denotes the physical position in megabases on the *Rattus *and *Mus *chromosomes [15, 16]. ** Markers beginning in PM_BW or contig are EST clones developed at the *Peromyscus *Genetic Stock Center (Weston Glenn et al., submitted BMC Genomics).

Our backcross panels facilitated linkage of markers at distances ranging from 1.2 cM to 35.8 cM. There are a few instances, however, where marker co-segregation occurs. These may be due to recombination "cold-spots", segmental inversions between the BW and PO strains, or simply the interval distance may be below our mapping panel resolution.

### Mapping of loci from *Rattus *Chrs 10 and 14

**Figure 1:**
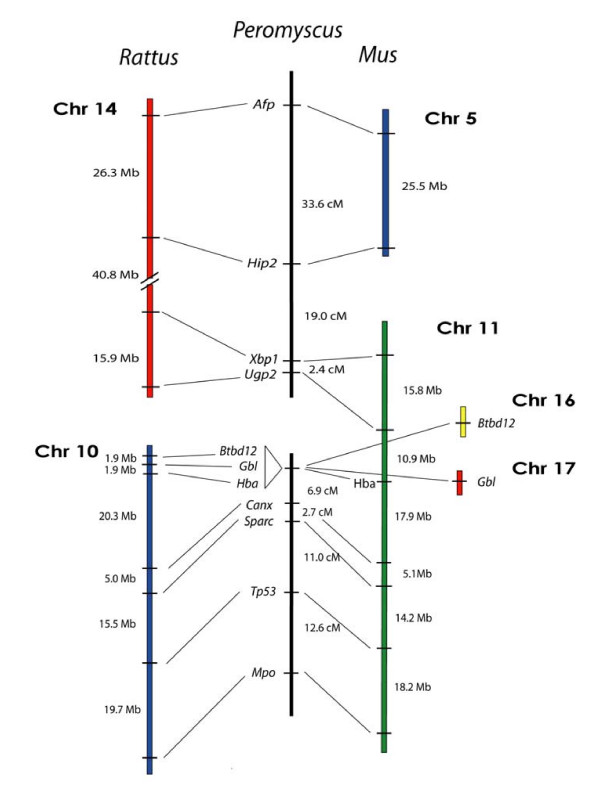
Comparison of the organization of genes on *Rattus *Chrs 14 and 10, and *Mus *Chrs 5, 11, 16, and 17, with the linkage of their orthologous genes in *Peromyscus*. The break in the continuity of the *Peromyscus *linkage groups indicates a lack of detectable linkage between the groups.

Our previous analysis of the *Peromyscus *genome using loci from *Mus *Chr 11 [17] indicated that there are two separate deer mouse linkage groups. ZOO-FISH data by Mlynarski et al. (submitted, BMC Evolutionary Biology) also supported this conclusion. These linkage groups correspond to *Rattus *Chrs 10 and 14 and the resulting chromosomal breakpoint is shared with the *Rattus *genome relative to the *Mus *genome (Figure 1). This breakpoint is also shared in other species, including human, chimpanzee, dog, and pig [11,18]. This conserved similarity led us to consider whether the deer mouse genome might share a higher degree of chromosomal similarity with *Rattus *than to *Mus*.

To explore this possibility, we expanded the existing linkage groups using loci whose orthologs lie on *Rattus *Chrs 10 and 14 but are not located on *Mus *Chr 11 (Figure 1). For the *Rattus *Chr 10 homology group, we generated markers for *Btbd12 *and *Gbl*, which correspond to regions of *Mus *Chr 16 and 17, respectively. Single loci for each segment were sufficient because the *Mus *Chrs 16 and 17 segments are small and most of *Rattus *Chr 10 is homologous with *Mus *Chr 11. We found both markers to be clearly linked to the terminal locus from *Rattus *Chr 10, *Xbp1*, with very high LOD scores (>30) indicating rat genome homology. However, all three markers co-segregated. At a distance of only 1.9 Mb in the *Rattus *genome, these markers are likely to be closer in the deer mouse than our panel is able to resolve.

Using a similar strategy, we also expanded the *Rattus *Chr 14 homology group using two loci from *Mus *Chr 5 that have orthologs on *Rattus *Chr 14, *Afp *and *Hip2*. Similar to both *Rattus *and *Mus *genomes, *Afp *and *Hip2 *are linked in the deer mouse at a distance of 33.6 cM (LOD = 4.4) (Figure 1). Similar only to the *Rattus *genome though, we found linkage in the deer mouse between *Hip2 *and the *Mus *Chr 11 marker *Xbp1 *at a distance of 19.0 cM (LOD = 11.1) (Figure 1). The marker intervals for this group were larger than those of the *Rattus *Chr 10 markers and allowed us to map 83.0 Mb (~82%) of *Rattus *Chr 14 in the deer mouse with only a few markers. Our results again show a greater similarity between *Peromyscus *genome organization and the *Rattus *genome than either to the *Mus *genome.

The high degree of *Rattus *genome similarity that we found for these two deer mouse linkage groups warranted examination of additional informative regions where synteny breakpoints occur between the *Mus *and *Rattus *genomes. While not comprehensive for every chromosome, this strategy accelerated our examination of chromosome evolution for the deer mouse genome and helped determine the best reference genome for future deer mouse genome mapping.

### Mapping the Informative Regions of *Rattus *Chr 1

**Figure 2:**
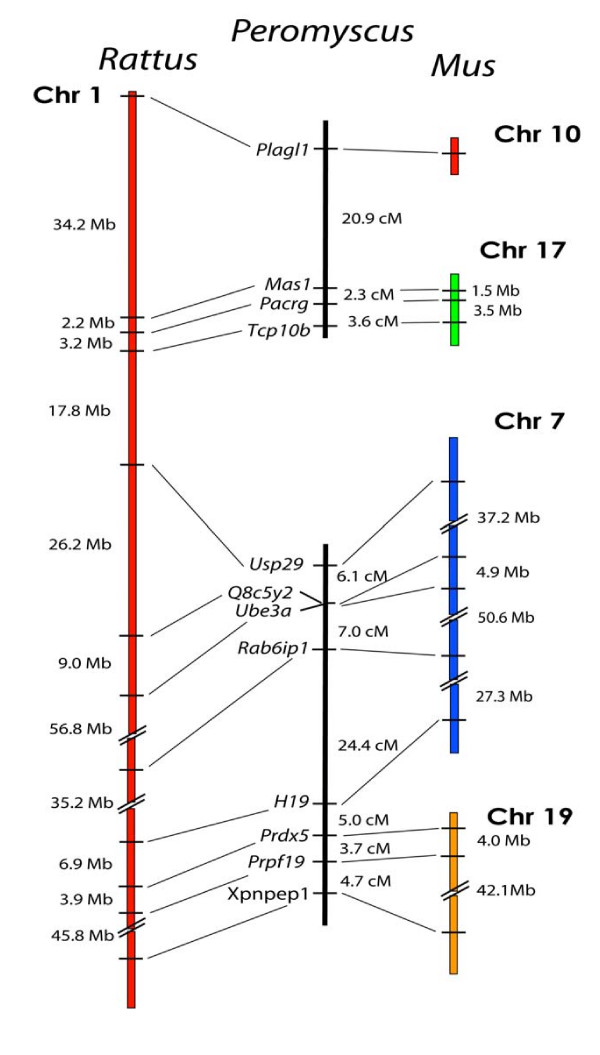
Comparison of the organization of genes on *Rattus *Chr 1, and *Mus *Chrs 7, 10, 17, and 19, with the linkage of their orthologous genes in *Peromyscus*. The break in the continuity of the *Peromyscus *linkage groups indicates a lack of detectable linkage between the groups.

We next chose markers that spanned *Rattus *Chr 1, focusing our efforts within the regions surrounding the breakpoints between *Mus *Chrs 7, 17, 10, and 19 (Figure 2). Within the *Mus *Chr 17 homology segment, we established linkage between *Tcp10b *and *Pacrg *at 3.6 cM and between *Pacrg *and *Mas1 *at 2.3 cM with LOD scores of 19.4 and 21.8, respectively. These distances are concordant with those for both the *Rattus *and *Mus *genomes. We had also previously shown linkage between *Tcp10b *and *Mas1 *using the deer mouse radiation hybrid panel [17]. We then genotyped the *Mus *Chr 10 marker *Plagl1 *to test for linkage to the *Mus *Ch17 markers and found *Mas1 *to be linked at a distance of 21.2 cM (LOD = 7.1). This linkage conservation is again consistent with the *Rattus *genome but not with *Mus*.

We next tested for linkage between the *Mus *Chr 17 and *Mus *Chr 7 homology segments of *Rattus *Chr 1 and found no linkage in *Peromyscus *between any *Mus *Chr 17 markers and the *Mus *Chr 7 marker *Usp29*, which is only 17.8 Mb away on *Rattus *Chr 1. We made no further efforts to extend the *Mus *Chr 7 linkage group, as *Usp29 *is only 5.93 Mb from the centromeric end of *Mus *Chr 7 and additional markers in this region were unlikely to yield a different result. We also tested for linkage between the *Mus *Chr 17 marker *Mas1 *and the *Mus *Chr 7 markers *Usp29 *and *Myod1 *on a PO × PO/BW backcross panel. This was to ensure that our negative linkage results were not a result of aberrant interspecific chromosomal recombination in the BW × BW/PO panel. Again, *Mas1 *did not link to either locus. The lack of linkage between *Mus *Chr 17 and *Mus *Chr 7 homology segments in the deer mouse genome constituted the first example of a common breakpoint between the deer mouse and *Mus *genomes when compared to *Rattus*.

Although uninformative for our comparative rearrangement analyses, we established linkage homology in the deer mouse for the large (~130 Mb) *Mus *Chr 7 section of *Rattus *Chr 1 using five markers: *Usp29, Q8C5Y2, Ube3a, Rab6ip1*, and *H19*. Althoughthe RIKEN cDNA marker *Q8C5Y2 *has not been accurately mapped in either *Rattus *or *Mus*, BLAST results indicated intervals between *Q8C5Y2 *and *Usp29 *at 26.4 Mb for the *Rattus *genome and 37.3 Mb in the *Mus *genome. In support of our placement, the *Mus *Chr 7/*Rattus *Chr 1 marker *Ube3a *co-segregated with *Q8C5Y2 *in the deer mouse. Our data for these five *Mus *Chr 7 markers showed high conservation of linkage and gene order with both *Rattus *and *Mus *genomes (Figure 2) with LOD scores all well above the 3.0 threshold. Our results for the *Mus *Chr 7 region were also concordant with a previous study [19] from which we utilized two of the same markers, *Usp29 *and *H19*.

We also found genome homology between *Rattus *and the deer mouse genome by markers spanning the breakpoint between the *Mus *Chr 7 and Chr 19 regions of *Rattus *Chr 1. The *Mus *Chr 7 marker *H19 *is linked to the *Mus *Chr 19 marker *Prdx5 *at a distance of 6.5 cM (LOD = 12.2). *Prdx5 *is also linked to a second *Mus *Chr 19 marker *Prpf19 *at a distance 4.9 cM (LOD = 13.4 cM).

Overall, our data for *Rattus *Chr 1 loci show that the two deer mouse linkage groups span two *Mus *genome breakpoints but only one *Rattus *genome breakpoint, which shows a continued bias towards similarity with the *Rattus *genome. Our results also imply that the *Mus *genome has been more rearranged in this region.

### Breakpoint Mapping of *Rattus *Chr 4 and Chr 6 Loci

To broaden the scope of the deer mouse map and help reduce bias resulting from any localized phenomenon such as segmental inversions, we acquired data for markers from multiple *Rattus *chromosomes. *Rattus *Chrs 4 and 6 were priority candidates because of the simple breakpoint arrangements and well-conserved gene orders between *Rattus *and *Mus*.

**Figure 3:**
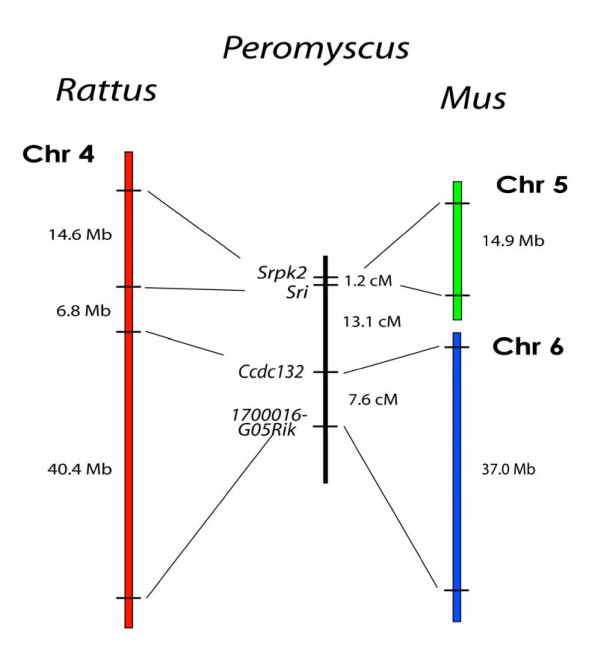
Comparison of the organization of genes on *Rattus *Chr 4, and *Mus *Chrs 5 and 6, with the linkage of their orthologous genes in *Peromyscus*.

*Rattus *Chr 4 is represented by the entirety of *Mus *Chr 6 and approximately 30 Mb of the centromeric end of *Mus *Chr 5. To test for a conserved organization in the deer mouse genome, we typed two markers from each side of the breakpoint on our backcross panel (Figure 3). We found the two *Mus *Chr 5 markers, *Srpk2 *and *Sri*, are linked to each other at 1.2 cM (LOD = 22.0) and the two *Mus *Chr 6 markers, *Ccdc132 *and *1700016G05Rik*, are linked to each other at a distance of 7.6 cM (LOD = 14.8). Spanning the breakpoint, we found that *Sri *and *Ccdc132 *are linked to each other at a distance of 13.3 cM (LOD = 10.3). Overall, our results span about 30% of *Rattus *Chr 4 and show conservation of the *Rattus *gene order. However, the linkage between *Sri *and *Srpk2 *was shorter than expected and may be due to a recombination cold-spot, an interspecific inversion, or a deletion.

**Figure 4:**
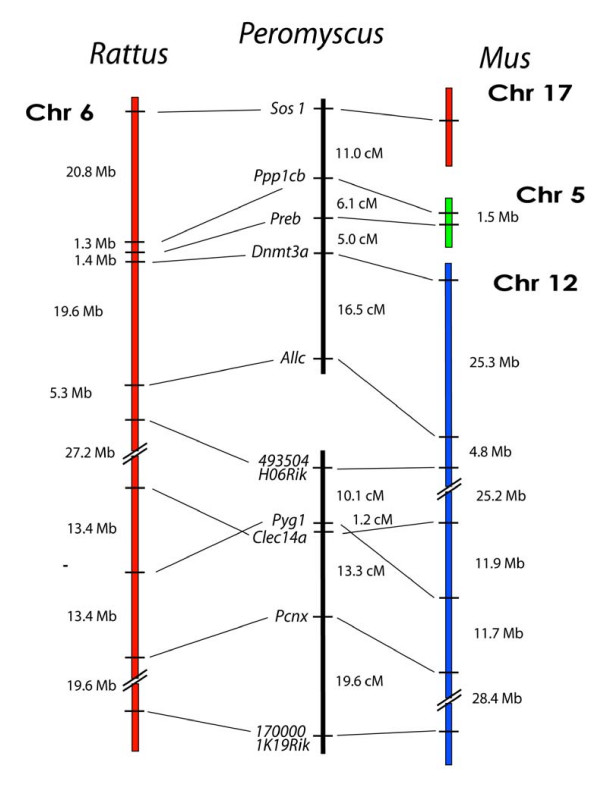
Comparison of the organization of genes on *Rattus *Chr 6, and *Mus *Chrs 5, 12, and 17, with the linkage of their orthologous genes in *Peromyscus*. The break in the continuity of the *Peromyscus *linkage groups indicates a lack of detectable linkage between the groups.

Our mapping of *Rattus *Chr 6 loci consisted of ten markers: *Sos1, Ppp1cb, Preb, Dnmt3a, Allc, 493504H06Rik, Pygl, Clec14a*, *Pcnx*, and *1700001K19Rik*. These markers span *Mus *chromosomes 17, 5, and 12 and our results yielded two separate linkage groups (Figure 4). The deer mouse linkage group homologous to the centromeric end of *Rattus *Chr 6 consists of five loci and represents three different chromosomes in *Mus*. This group is conserved in both synteny and gene order with the *Rattus *genome. *F*rom *Mus *Chr 17, *Sos1 *is linked to the *Mus *Chr 5 marker *Ppp1cb *at a distance of 11.0 cM (LOD = 12.6). Within the *Mus *Chr 5 segment, *Ppp1cb *is linked to *Preb *at a distance of 6.1 cM (LOD = 16.8). A second *Mus *breakpoint is spanned by the linkage between *Preb *and the *Mus *Chr 12 marker *Dnmt3a *at a distance of 5.2 cM (LOD = 16.4). Also from *Mus *Chr 12, *Allc *represents the terminal marker and links to *Dnmt3a *at an interval of 16.5 cM (LOD = 7.6).

The five remaining markers from *Mus *Chr 12 form a second linkage group in *Peromyscus*. Although synteny with both *Rattus *Chr 6 and *Mus *Chr 12 is conserved, we discovered an inversion with respect to both *Mus *and *Rattus *involving markers *Clec14a *and *Pygl *(Figure 4). Defining this inversion, *Pygl *is linked to *493504H06Rik *at a distance of 4.2 cM (LOD = 9.2) while *Clecl4a *is linked to the more telomeric marker *Pcnx *at a distance of 18.2 cM (LOD = 6.3). We also found that *Clec14a *and *Pygl *have smaller distance interval at 1.3 cM (LOD = 21.2) than would have been expected from the physical intervals in *Mus *(11.9 Mb) and *Rattus *(13.4 Mb). Forming the end of the linkage group, *Pcnx *was linked to *1700001K19Rik *at a distance of 25.3 cM (LOD = 3.9).

The spanning of two *Mus *genome breakpoints by the deer mouse linkage groups again indicates a more *Rattus*-like genome organization. However, the breakpoint between the two *Peromyscus *linkage groups flanked by the two *Mus *Chr 12 markers *Allc *and *493504H06Rik *represents a unique rearrangement that differs from both the *Rattus *and *Mus *genomes. ZOO-FISH results from Mlynarski et al. (submitted, BMC Evolutionary Biology) also concur that *Rattus *Chr 6 is indeed represented by two separate chromosomes in the deer mouse.

### Mapping Loci from *Mus *Chr 1/*Rattus *Chrs 5, 9, and 13

**Figure 5:**
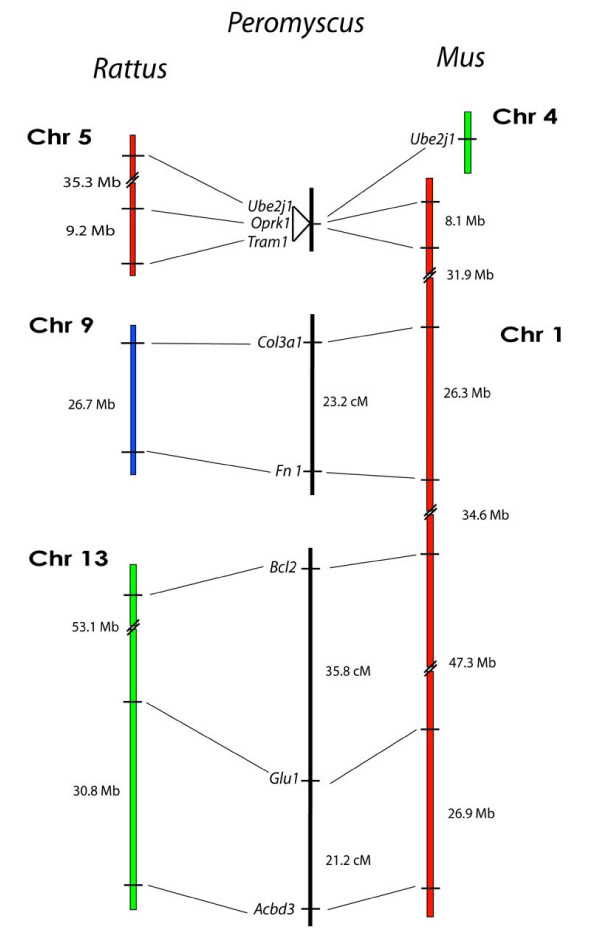
Comparison of the organization of genes on *Rattus *Chrs 5, 9, and 13, and *Mus *Chrs 1 and 4 with the linkage of their orthologous genes in *Peromyscus*. The break in the continuity of the *Peromyscus *linkage groups indicates a lack of detectable linkage between the groups.

To avoid possible bias towards finding only *Rattus *genome similarity, we also selected markers to span *Rattus *synteny breakpoints in relation to the *Mus *genome. This involved markers that span approximately 89% of *Mus *Chr 1 but are located separately in *Rattus *on Chrs 5, 9, and 13 (Figure 5). With exception of the *Rattus *Chr 9 marker *Col9a1*, we found linkage between all of the markers within their *Rattus *chromosome homology groups but not between them, indicating a bias toward *Rattus *genome similarity.

For the *Rattus *Chr 5 region, we detected non-segregating linkage between *Oprk *and *Tram *in the deer mouse with a strong LOD score of 20.8. To further investigate *Rattus *genome homology, we employed a third *Rattus *Chr 5 marker, *Ube2j1*, which is located on *Mus *Chr 4. Consistent with *Rattus *genome organization, *Ube2j1 *linked strongly (LOD = 23.5) but without segregation to *Oprk1 *and *Tram1*, despite a distance 35.3 Mb in the *Rattus *genome between *Ube2j1 *and *Oprk1*.

At the telomeric end of *Mus *Chr 1, we detected linkage between the *Rattus *Chr 13 markers *Acbd3 *and *Glul *at a distance of 21.2 cM (LOD = 9.6) and between *Glul *and *Bcl2 *at 35.8 cM (LOD = 3.8) (Figure 5). However, we did not detect linkage between *Bcl2 *and the *Mus *Chr 9 marker *Fn1*, as would be expected by *Mus *genome homology.

**Figure 6:**
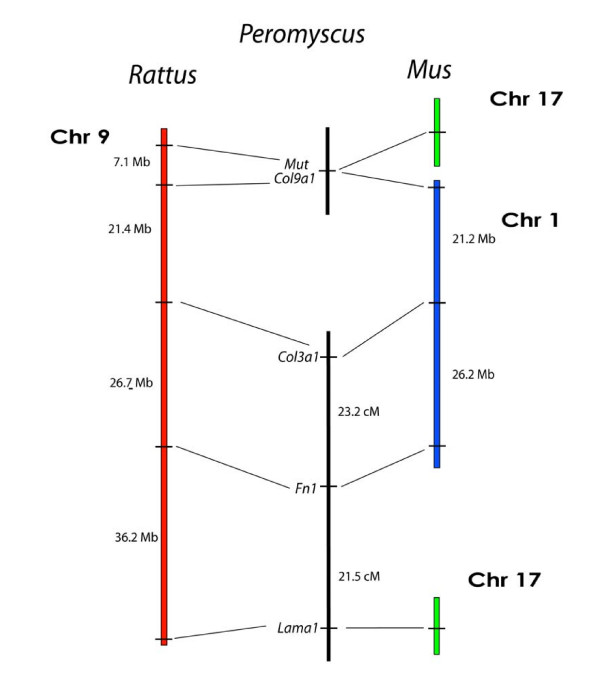
Comparison of the organization of genes on *Rattus *Chr 9, and *Mus *Chrs 1 and 17, with the linkage of their orthologous genes in *Peromyscus*. The break in the continuity of the *Peromyscus *linkage groups indicates a lack of detectable linkage between the groups.

Amongst the *Rattus *Chr 9 markers, we found *Col3a1 *and *Fn1 *were linked at a distance of 18.8 cM (LOD = 10.3). However, *Col3a1 *is surprisingly not linked to *Col9a1*, which represents a deviation from the *Rattus *genome. To confirm these results, we also tested *Mut*, a marker that is closely linked to *Col9a1 *on *Rattus *Chr 9 (Figure 6). *Mut *is located 7.13 Mb from *Col9a1 *on *Rattus *Chr 9 but in *Mus *is located separately on Chr 17. *Mut *did not link to *Col3a1 *but did co-segregate with *Col9a1 *(LOD = 12.0), thus identifying a linkage similarity between the *Rattus *and deer mouse genomes. The breakpoints present in the deer mouse and *Mus *maps for this region are offset and may represent breakpoint area re-usage and a rearrangement hotspot [13]. However, more detailed mapping using markers located between *Col9a1 *and *Col3a1 *on *Rattus *Chr 9 are needed to refine the breakpoint location. We discovered additional *Rattus *Chr 9 similarity using the marker *Lama*, which represents a second and separate region of *Mus *Chr 17 than that of *Mut *(Figure 6). We found *Lama1 *and *Fn1 *are linked in the deer mouse at a distance of 32.7 cM (LOD = 4.7).

### Mapping Loci from *Mus *Chr 8/*Rattus *Chrs 16, 17, and 19

**Figure 7:**
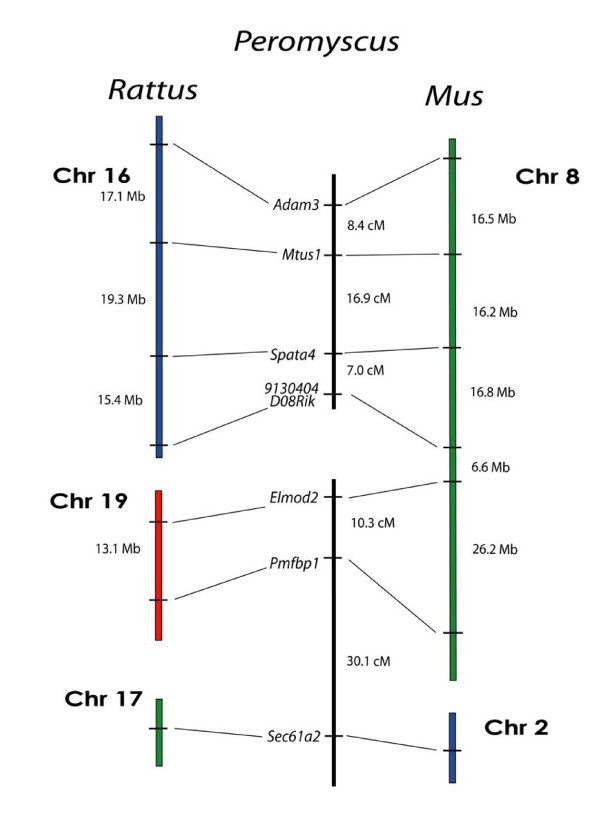
Comparison of the organization of genes on *Rattus *Chrs 16, 17, and 19, and *Mus *Chrs 2 and 8, with the linkage of their orthologous genes in *Peromyscus*. The break in the continuity of the *Peromyscus *linkage groups indicates a lack of detectable linkage between the groups.

As another test of *Peromyscus *genome homology to *Mus*, we performed an analysis using six loci from *Mus *Chr 8 that form two linkage groups in the *Rattus *genome (Figure 7). We selected markers in each group that are less than 20.Mb apart in the *Rattus *genome to facilitate deer mouse linkage detection. Additionally, the two markers that flank the breakpoint, *9130404H06Rik *and *Elmod2*, are about 16.0 Mb apart in *Mus*, which is well within the range of our mapping panel.

As with the *Mus *Chr 1 analysis, we found linkage with highly significant LOD scores but only within the individual *Rattus *chromosomal groups, not between them. For the *Rattus *Chr 16 segment, *Adam3 *and *Mtus1 *are linked at distance of 8.4 cM (LOD = 14.8) and *Mtus1 *is linked to *Spata4 *at a distance of 16.9 cM (LOD = 9.3). *Spata4 *is also linked to the terminal marker *9130404D08Rik *at a distance of 7.0 cM (LOD = 16.4). The two markers from *Rattus *Chr 19, *Elmod2 *and *Pmfbp1*, are linked 10.3 cM (LOD = 12.5).

Based on suggestions from ZOO-FISH results (Mlynarski et al., submitted BMC Evolutionary Biology), we also examined an additional chromosomal segment for linkage. Representing *Rattus *Chr 17 and *Mus *Chr 2, *Sec61a2 *links to the *Rattus *Chr 19/*Mus *Chr 8 marker *Pmfbp1 *at a distance of 30.1 cM (LOD = 3.7). This linkage indicates a clear deviation from both the *Rattus *and *Mus *genomes by the deer mouse genome and highlights the benefit of performing cytogenetic analyses in tandem with meiotic linkage mapping.

### Linkage Testing of *Mus *Chrs 17, 5, and 13 Loci

**Figure 8:**
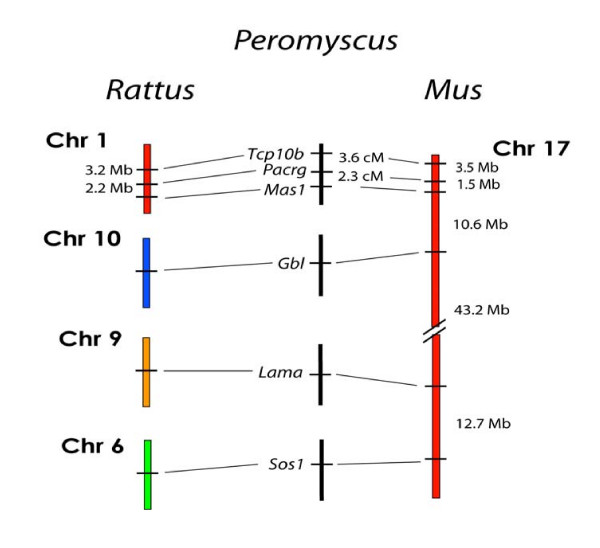
Comparison of the organization of genes on *Rattus *Chrs 1, 6, 9, and 10, and *Mus *Chr 17 with the linkage of their orthologous genes in *Peromyscus*. The break in the continuity of the *Peromyscus *linkage groups indicates a lack of detectable linkage between the groups.

**Figure 9:**
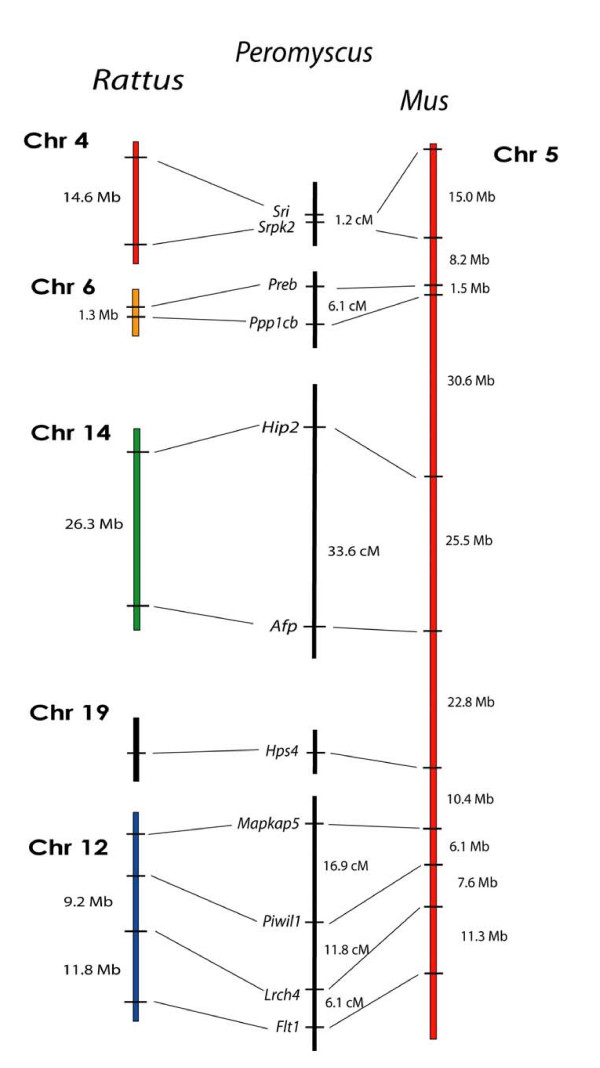
Comparison of the organization of genes on *Rattus *Chrs 4, 6, 12, 14, and 19, and *Mus *Chr 5 with the linkage of their orthologous genes in *Peromyscus*. The break in the continuity of the *Peromyscus *linkage groups indicates a lack of detectable linkage between the groups.

**Figure 10:**
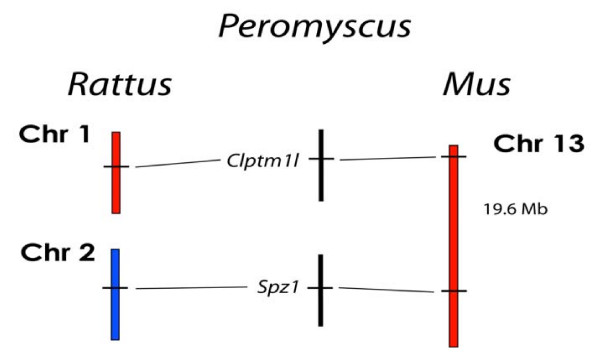
Comparison of the organization of genes on *Rattus *Chrs 1 and 2, and *Mus *Chr 13, with the linkage of their orthologous genes in *Peromyscus*. The break in the continuity of the *Peromyscus *linkage groups indicates a lack of detectable linkage between the groups.

We performed additional tests for *Mus *genome similarity within the deer mouse using loci from *Mus *Chrs 17, 5, and 13 (Figures 8, 9, and 10). These results also came out negative for *Mus *genome similarity. *Mus *chromosomes 17 and 5 are two of the most rearranged chromosomes in the *Mus *genome compared to the *Rattus *genome. *Mus *Chr 17 and has seven different regions representing five different *Rattus *chromosomes (Figure 8) and *Mus *Chr 5 has four major regions representing four *Rattus *chromosomes and three very small segments representing three additional *Rattus *chromosomes (Figure 9). Positive linkage results for these highly rearranged chromosomes in the deer mouse would have been a strong indicator of *Mus *homology.

For *Mus *Chr 17, six markers (*Tcp10b, Mas1*, and *Pacrg *from *Rattus *Chr 1; *Gbl *from *Rattus *Chr 10; *Lama1 *from *Rattus *Chr 9; and *Sos1 *from *Rattus *Chr 6) were tested for linkage in all possible arrangements despite having already been assigned to other deer mouse linkage groups. No linkage was found other than that which already existed amongst the *Rattus *Chr 1 markers *Tcp10b, Pacrg*, and *Mas1 *(Figure 8).

We conducted a similar test for several markers from *Mus *Chr 5 (Figure 9). Eleven markers representing five *Rattus *chromosomes were tested for linkage. As in the *Mus *Chr 17 analysis, linkage was found only between markers located on the same *Rattus *chromosomes.

Two *Mus *Chr 13 markers, *Clptml1 *and *Spz*, also failed to show linkage despite being only about 19.6 Mb apart in the Mus genome, thus further reinforcing the linkage group disparities between the deer mouse and *Mus *genomes. *Clptml1 *is located in *Rattus *Chr 1 and *Spz1 *is on *Rattus *Chr 2 (Figure 10).

## Conclusion

### Genome Mapping and Genomic Evolution

Development and availability of multiple mapping tools is essential for accurate and timely exploration of any species' genome. Three methods have already been employed in mapping small portions of the deer mouse genome in the form of cytogenetics [14,20], meiotic segregation analysis [17,21-25], and a whole-genome radiation hybrid cell panel [17]. These tools are most powerful when used in combination, as exemplified by Rowe et al. [2] for *Mus *and by Menotti et al. [26] for the cat.

Here we significantly expand the *Peromyscus *meiotic segregation mapping data using two *P. maniculatus *× *P. polionotus *interspecific backcross panels and present the most comprehensive comparative linkage mapping data for the deer mouse to date using Type I gene markers. In addition to providing an important genetic tool for *Peromyscus *research, we tested whether the deer mouse genome displayed organizational homology to that of *Mus musculus*, *Rattus norvegicus*, or a combination of both. Our results indicate a large degree of gene order and synteny conservation by the deer mouse genome with that of *Rattus*.

Our analysis was done by establishing linkage over approximately 35% of the deer mouse genome using gene markers that predominantly spanned junctions of large-scale genome rearrangements between the *Rattus *and *Mus *genomes. While using the *Rattus *genome as the reference, we tested 13 *Mus *genome breakpoints. Ten of the 13 breakpoints spanned by the *Rattus *genome were similarly linked in the deer mouse genome. In contrast, only one of 12 *Rattus *genome breakpoints that we examined while using the *Mus *genome as a reference closely coincided with any linkage breakpoints that we found in deer mouse genome. These data demonstrate that the organization of the deer mouse and *Rattus *genomes are more similar to each other than either is to *Mus*.

There are three instances, however, where the deer mouse map differs from both the *Rattus *and *Mus *maps. Two examples are located between markers *Allc *and *493504H06Rik *(Figure 4) and between markers *Col9a1 *and *Col3a1 *(Figure 6). Approximately 21 Mb separates the latter pair in both *Rattus *and *Mus*, so additional markers will need to be applied to the deer mouse panel to better pinpoint the location of this breakpoint. The third instance is the unique deer mouse linkage of *Sec61a2 *to *Pmfbp1*. Collaborative efforts have also helped to inform, as well as confirm, some of these data using additional tools such as ZOO-FISH analyses using flow-sorted whole chromosome probes (Mlynarski et al., submitted BMC Evolutionary Biology). The strong organizational similarity of the deer mouse genome with the *Rattus *genome rather than the more morphologically similar *Mus musculus *suggests that a significant amount of rearrangement occurred in the *Mus *genome after the divergence of the cricetid and murid lineages. Concomitantly, our results suggest that the genomic organizations of *Rattus *and *Peromyscus *are more representative of the ancestral muroid genome than the *Mus *genome, which is in agreement with previous literature that indicated a higher rate of genome rearrangement for *Mus *[5,6]. Most eutherian genomes have 30 to 40 blocks of homology with the human genome while the *Mus *genome is extraordinary with approximately 200 homology blocks. However, the *Mus *genome is not unique in having higher relative rearrangement rates, as the canine and gibbon genomes have approximately twice the average number of homology blocks [27].

Our results also show that genome rearrangement can act independently from other forms of genome evolution, such as sequence mutation. Although rodent sequence mutation rates are higher compared to other mammals, such measurements of genome evolution show *Mus *and *Rattus *shared a common ancestor significantly more recently than either have with *Peromyscus*. Our data does not propose to change this phylogeny but rather merely highlights that DNA sequence variation and chromosome rearrangement are independent processes and greater understanding of both processes can provide different insights into the evolution of the structure and function of the eukaryotic genome.

## Methods

### Development of a *P. maniculatus *Backcross Panel

We chose interspecific backcross analysis in order to maximize genetic polymorphism and for the ease of linkage analysis [28]. The two species used in the cross were the deer mouse (*P. maniculatus bairdii; *BW) and the old field mouse (*P. polionotus subgriseus; *PO) and were obtained from the *Peromyscus *Genetic Stock Center at the University of South Carolina [29]. We set up the initial crosses in only one direction, BW females × PO males, to generate interspecific hybrid F1's. The direction of this cross is essential, as the reciprocal cross results in lethal overgrowth of the offspring [30].

We created two separate backcross panels, BX116 and BX2, for the linkage analysis. For the BX116 panel, we bred twelve hybrid (*plt *BW × PO) F1 animals with 12 unrelated *plt *BW animals to generate 116 backcross progeny. Backcrosses to BW can be performed using both female and male F1 hybrid animals, as both matings will give viable offspring. However, all but one of the matings used for this panel were F1 × BW (♀ × ♂).

We used a similar strategy for the BX2 panel but the *plt *BW stock was substituted with wild-type BW stock. The *plt *allele originated in a different subspecies of *P. maniculatus *than the BW stock to which it was crossed. This additional backcross panel was designed to circumvent intraspecific SNP variation and possible recombination problems due to chromosomal inversions that are known to exist within some *P. maniculatus *sub-species.

To create the BX2 panel, we crossed four F1 males from separate unrelated ♀ BW × ♂ PO matings to non-sibling BW females, which were generated from separate matings. This resulted in four unrelated backcross families. We obtained twenty-two ♀ BW × ♂ F1 offspring from each of three of these backcross matings and 20 offspring were obtained from the fourth for a total of 86 backcross animals. These were grouped in a 96-well tray along with the eight parentals and a positive and a negative control. We employed this strategy to minimize variation within families while maximizing information between families. Similarly, the strategy of crossing F1 hybrid males with BW females minimized variation due to gender-based differences in recombination frequency.

We extracted genomic DNA from all backcross parents and progeny for the BX116 panel from 1.0 cm tail snips with the Qiagen DNEasy Tissue Kit using the manufacturers protocol (Qiagen, Inc.). Genomic DNA for the BX2 panel animals was extracted using a phenol/chloroform/isoamyl alcohol extraction method to increase yields.

### Marker Development

We obtained primer sequences for some Type I markers from published sets of orthologous gene markers. These are termed UMPS, CATS, and TOASTs [31-33]. Primer sequences for *Il23a, Mas1, H19, Sos1*, and *Grb10 *were developed or obtained from published *Peromyscus *data [34-36]. *Trp53 *primers were developed from *P. maniculatus *sequence and were kindly provided by Michael McLachlan. We developed all other primers from *P. maniculatus *EST sequences (Weston Glenn et al., submitted BMC Genomics). Deer mouse EST clones were used for marker design because of greater PCR amplification success (>80% versus ~60% for the published sets).

We designed all the markers to be ~400 bp–1500 bp and to span an intron to increase polymorphism detection. This was done by aligning deer mouse DNA sequences to *Mus musculus *genomic sequences using cross-species megaBLAST (NCBI). Some critical markers however, spanned larger introns and resulted in amplicons slightly larger than the ideal size parameters.

PCR cycling conditions for all Type I markers were optimized for *P. maniculatus *and *P. polionotus *DNA using a MJ Research PTC-200 DNA Engine gradient thermal cycler and are defined as follows: **1) **Standard: 95°C for 14.5 min followed by 35 cycles of (95°C for 30 s, 48–65°C for 30 s, 72°C for 30 s per 0.5 kb), 72°C for 10 min, 4°C hold. **2) **Touchdown65 (TD65): 95°C for 14.5 min followed by 20 cycles of (95°C for 30 s, 65°C for 30 s minus 0.5°C/cycle for 20 cycles, 72°C for 30 s per 0.5 kb) followed by 15 cycles of (95°C for 30 s, 55°C for 30 s, 72°C for 30 s per 0.5 kb), 72°C for 10 min, 4°C hold. If no product was obtained using Touchdown65, the starting annealing temperature was changed to 60°C or 55°C, with the final annealing temperature remaining 10°C lower than the starting temperature.

PCR was performed using 20 ng of genomic DNA in a 10 μl reaction containing 1 μl 10× Qiagen HotStar buffer (1.5 mM MgCl_2_), 200 μM each dNTP, 0.4 μM forward primer, 0.4 μM reverse primer, and 1 unit Qiagen HotStar Taq polymerase. Some difficult templates required the use of Qiagen Q-solution at either 1× or 0.5× strength. Four markers, *Sparc, Xbp1, Grb10*, and *Ugp2 *required 2.0 mM MgCl_2_.

For all markers, 5 μL of each amplification product was visualized by gel electrophoresis. The remaining 5 μL portion of the PCR products was treated for sequencing with 5 units Exonuclease I and 0.75 units Shrimp Alkaline Phosphatase (SAP) and incubated at 37°C for 15 minutes followed by heat-inactivation at 80°C for 15 minutes. For sequencing reactions, 2.0μL of purified PCR product was direct sequenced with BigDye (v3.1) (ABI) on an ABI 3130 × l according to the manufacturer's protocol.

Sequence identities were verified by cross-species megaBLAST or BLASTN search to the *Mus musculus *genome. Any predicted simple size polymorphisms between BW and PO were tested by gel electrophoresis using amplification products from BW, PO, and BW/PO mix (equal ratio) DNAs. Markers not showing size polymorphisms were further analyzed for species-specific RFLPs by sequence comparison using Sequencher software (Gene Codes Corporation), the TCAG program available as part of the Biology Workbench software utilities provided at the San Diego Supercomputer Center [37], or with the SNP-RFLPing program [38]. Candidate enzymes were chosen from those predicted by the software. RFLP tests for each marker and enzyme were conducted according to manufacturer's protocols on 10 μL PCR products from a template test panel consisting of DNA from BW, PO, a BW/PO mix (equal ratio), and a negative using only TE. All RFLP products were analyzed by gel electrophoresis. Any essential markers that could not be genotyped by either size polymorphism or RFLP were sequenced on all the backcross animals and their parents.

### PCR Typing of the Backcross Panel

We tested the backcross panel parental mice with each marker prior to use on the backcross panel. We typed then typed the markers on all possible backcross animals whose parents exhibited the expected genotype. On the BX116 panel, no fewer than 73 animals were used for data to establish linkage between any two markers with exception of *Gbl *to *Hba *and *Hba *to *Canx*, of which both only used 39 animals due to the small usable data set for *Hba*. For the BX2 panel, no fewer than 60 animals were typed between any two markers with the exception of *Mut *and *Col9a1*, for which only 40 animals could be genotyped in common.

### Data Analysis

We performed backcross linkage analysis using Map Manager QTX software [39]. A minimum LOD score of 3 was used to establish linkage. Ordering of markers typed on the backcross data was determined by subjecting the data to the "ripple test", which evaluated local permutations and selected the optimal order based on minimum breakage. Once linkage and gene order was established with high degree of confidence, omitted or unavailable genotypes could sometimes be inferred by Map Manager, as double crossovers between closely linked markers are rare. The procedure of inferring genotypes did not change any gene orders but typically tightened linkages slightly and raised LOD scores.

## Abbreviations

BLAST Basic Local Alignment Search Tool

BW *Peromyscus maniculatus bairdii*

CATS Comparative Anchor Tag Sequences

cDNA complementary DNA

Chr chromosome

cM centimorgan

ddH_2_0 distilled deionized water

dNTP dinucleotide triphosphate

EDTA ethylenediaminetetraacetic acid-disodium salt

EST expressed sequence tag

LOD logarithm of the odds (to the base 10)

Mb megabase

mya million years ago

NEB New England Biolabs

PCR polymerase chain reaction

*plt *platinum coat-color mutation

PO *Peromyscus polionotus subgriseus*

RFLP restriction fragment length polymorphism

SAP shrimp alkaline phosphatase

SNP single nucleotide polymorphism

SSLP simple sequence length polymorphism

TBE Tris-Borate EDTA

TE Tris-EDTA (10 mM Tris, 1 mM EDTA)

TLE Tris-low EDTA (10 mM Tris, 0.1 mM EDTA)

TOAST traced orthologous amplified sequence tags

UMPS universal mammalian primer sequence

ZOO-FISH cross-species fluorescence in-situ hybridization

## Authors' contributions

CR was the lead researcher, conceived the study, participated in its design and coordination, developed markers and assays, participated in the design of the backcross panel, performed linkage analysis, performed molecular genetic experiments, performed sequence alignments, and drafted the manuscript. AL developed markers and assays, performed molecular genetic experiments, and performed sequence alignments. JG developed markers and assays, participated in the design of the backcross panels, performed molecular genetic experiments, performed sequence alignments, and edited the manuscript. PV developed markers and assays, performed linkage analyses, participated in the design of the study, and contributed to and edited the manuscript. RO participated in the design and coordination of the study and helped to draft the manuscript. MD was the principle investigator on the project, participated in its design and coordination, performed sequence alignments, participated in the design of the backcross panels, and contributed in drafting the manuscript and figures. All authors read and approved the final manuscript.
